# Axial Force Transmission Through Orthosis Straps in a Neonatal Hip Flexion–Abduction Orthosis: An Exploratory In Vivo Study in Infants with Developmental Dysplasia of the Hip

**DOI:** 10.3390/children13060777

**Published:** 2026-06-02

**Authors:** Paul Schwanitz von Keitz, Kira Henriette Liebau, Wolfram Mittelmeier, Susanne Froehlich

**Affiliations:** 1 Rehafachzentrum Bad Füssing, Orthopädische Klinik, 94072 Bad Füssing, Germany; 2 Orthopedic Department, University Medicine Rostock, 18057 Rostock, Germany

**Keywords:** developmental hip dysplasia, treatment, axial load, biomechanics, orthosis

## Abstract

**Highlights:**

**What are the main findings?**

- The Mittelmeier–Graf hip flexion–abduction orthosis exerts a low total axial load of approximately 5–12% of the newborn’s body weight, regardless of the adjustment setting.
- Asymmetrical adjustment redistributes load between sides without significantly altering total axial force.

**What are the implications of the main findings?**

- The study demonstrates that axial loading in the hip flexion–abduction orthosis is low, indicating a favorable biomechanical safety profile in neonates.
- Despite the biomechanical efficacy of the asymmetrical setting, the symmetrical application may be clinically preferable due to improved parental compliance and avoidance of postural asymmetry.

**Abstract:**

**Background:** Developmental dysplasia of the hip (DDH) is the most common congenital musculoskeletal disorder in newborns. Flexion–abduction orthoses are widely used in early treatment; however, in vivo data on their biomechanical load characteristics remain limited. This study aimed to evaluate axial force transmission in a hip flexion–abduction orthosis and to compare load patterns between healthy newborns and infants with DDH. **Methods:** In this exploratory observational study, 36 newborns (19 healthy, 17 with unilateral DDH) were examined within the first week of life. Axial forces transmitted through a Mittelmeier–Graf hip flexion–abduction orthosis (MGO) were measured using integrated force sensors under symmetrical and asymmetrical adjustment configurations. Intergroup comparisons were performed using non-parametric statistical tests. **Results:** Mean axial forces were significantly higher in healthy infants than in those with DDH under both symmetrical (4.02 N vs. 2.51 N; *p* = 0.019) and asymmetrical (3.67 N vs. 1.83 N; *p* = 0.001) conditions. Relative load corresponded to approximately 11–12% of body weight in healthy infants and 5–7% in the DDH group. No significant intra-individual differences were observed between dysplastic and contralateral hips. Orthosis configuration (symmetrical vs. asymmetrical) did not significantly affect load distribution. **Conclusions:** This exploratory in vivo study demonstrates that axial load transmission in a hip flexion–abduction orthosis is low and influenced by underlying hip pathology. Infants with DDH generate lower forces than healthy newborns, potentially reflecting altered biomechanics. As no significant differences were observed between orthosis configurations, symmetrical adjustment may be favored in clinical practice due to better usability and compliance. Further studies with larger cohorts are needed to confirm these findings.

## 1. Introduction

Developmental dysplasia of the hip (DDH), with a global incidence of 2–4% in newborns, is the most common congenital musculoskeletal disorder [1,2,3,4]. Screening and classification are commonly performed using the Graf ultrasound method, which remains the international diagnostic standard for neonatal hip dysplasia [5,6]. Dysplastic hip joints require postnatal maturation of the acetabulum [7,8]. Conservative treatment using flexion–abduction orthoses remains the standard treatment for early stages of DDH and has demonstrated favorable clinical outcomes in multiple studies [9,10]. In cases of unilateral DDH, this approach also restricts the position of the unaffected hip. Altered muscle biomechanics and modified activation patterns have been described in patients with dysplastic hips, potentially affecting the mechanical environment within orthotic systems [11,12]. Computational biomechanical methods have increasingly been used to analyze the mechanical environment of dysplastic hips during treatment. The duration and outcome of conservative DDH treatment depend on multiple clinical factors. Recent clinical analyses have demonstrated that parameters such as the age at treatment initiation, the acetabular alpha angle, and bilaterality significantly influence treatment duration and outcome [13]. These findings highlight the multifactorial nature of DDH therapy and emphasize that biomechanical aspects of orthotic treatment represent only one component of the overall therapeutic strategy. Biomechanical investigations have demonstrated that the design of flexion–abduction orthoses substantially influences axial load transmission in infants treated for DDH [14,15]. Computational biomechanical models have further shown that orthotic treatment modifies joint reaction forces and the mechanical environment of the dysplastic hip joint, thereby facilitating reduction and stabilization of the femoral head during treatment [16,17]. The magnitude and distribution of forces transmitted through neonatal hip orthoses may be clinically relevant because they potentially influence therapeutic hip positioning, stabilization of the femoral head, patient comfort, neuromuscular loading, and parental acceptance of the device. Axial force transmission within hip flexion–abduction orthoses may be biomechanically relevant because these forces contribute to maintenance of therapeutic hip positioning and may indirectly influence femoral head stabilization within the acetabulum during treatment. In addition, excessive or asymmetrical force transmission may affect patient comfort, neuromuscular activation patterns, handling of the orthosis, and parental compliance, all of which are clinically relevant determinants of successful conservative DDH treatment. Therefore, characterization of orthosis-related force transmission may improve understanding of the mechanical environment generated during conservative DDH management. Excessive or asymmetrical force transmission may negatively affect handling and compliance, whereas insufficient stabilization could theoretically reduce therapeutic effectiveness. Therefore, improved understanding of orthosis-related force transmission may contribute to optimization of conservative DDH treatment strategies. Although hip flexion–abduction orthoses are widely used in the conservative treatment of developmental dysplasia of the hip (DDH), quantitative in vivo data regarding biomechanical load transmission within these orthotic systems remain very limited. Previous studies have primarily focused on radiological outcomes, treatment success rates, and clinical positioning concepts, whereas the dynamic mechanical interaction between the infant and the orthosis has rarely been investigated directly. Consequently, little is known about the magnitude and distribution of forces transmitted through neonatal hip orthoses during physiological infant movement.

The present exploratory study therefore aimed to provide first in vivo biomechanical measurements of axial force transmission within an MGO in infants with and without DDH. By directly quantifying force transmission under clinical conditions, this study seeks to contribute novel biomechanical information relevant to orthosis design, treatment monitoring, and conservative DDH management.

**Figure 1:**
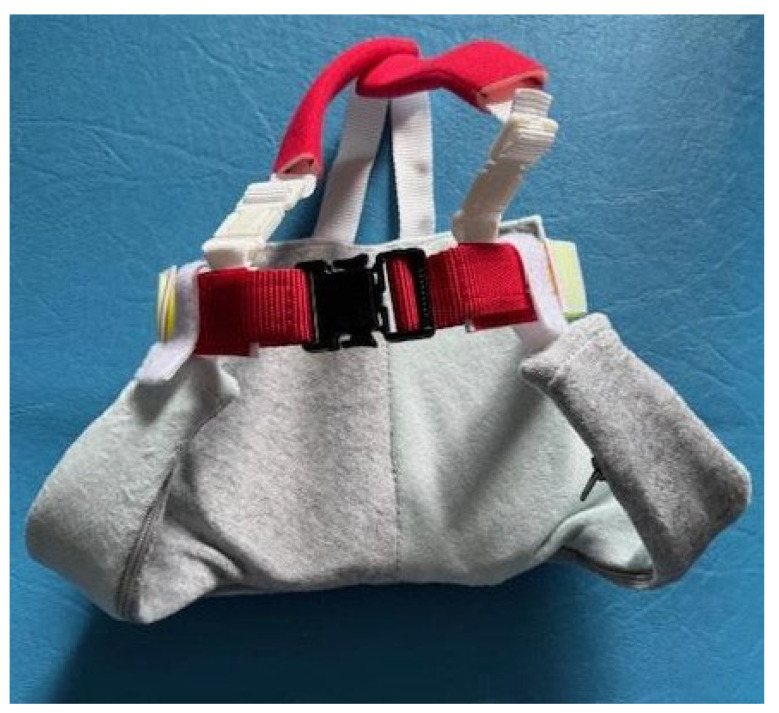
Mittelmeier–Graf hip flexion–abduction orthosis.

**Figure 2:**
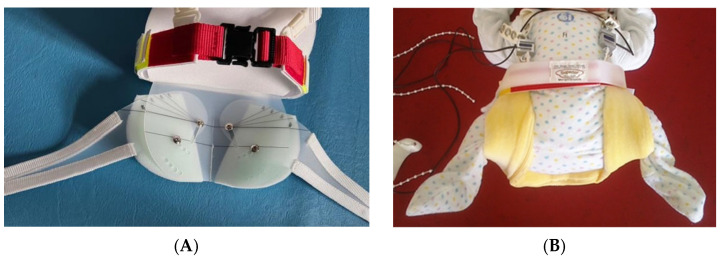
Orthosis with symmetrically adjusted leg components (**A**); symmetrical adjustment with measurement sensors positioned at the front (**B**).

**Figure 3:**
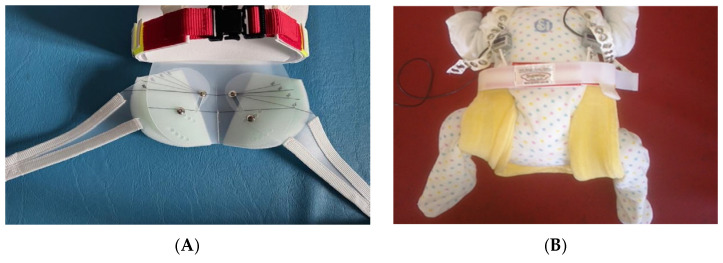
Orthosis with asymmetrically adjusted leg components (**A**); asymmetrical adjustment with measurement sensors positioned at the front (**B**).

The MGO (Figure 1)was specifically designed to maintain the natural positioning of the unaffected hip. Critically, prior studies have not differentiated between the forces generated by healthy infants and by those with DDH. Consequently, it remains unclear how the pathophysiological mechanisms associated with dysplastic hips influence the mechanical environment within the orthosis. The present study aims to address this gap by directly comparing these two cohorts. The MGO is a modification of the Ideal Spreader Pants and has been clinically applied for the treatment of DDH under ultrasound monitoring [18]. Due to its specific design, it exhibits a distinct force distribution pattern [18]. The orthosis consists of adjustable leg supports connected to a pelvic strap, which transitions dorsally into a pelvic base plate. Two support wedges are incorporated to prevent contraindicated overabduction. Axial forces are primarily transmitted via the pelvic strap and base plate, whereas the shoulder straps mainly serve to prevent displacement of the device [14]. The pelvic plate is perforated and allows attachment of the leg supports using adjustable screws, enabling variation in leg length and hip flexion angle. Importantly, the orthosis can be configured asymmetrically. Under symmetrical configuration (Figure 2), both hips were positioned according to the standard therapeutic settings of the MGO with clinically standardized flexion and abduction positioning (therapeutic squatting–spreading “human position”). In asymmetrical configurations (Figure 3), selective abduction adjustment was applied to the affected side while maintaining physiological positioning of the unaffected hip. Rotational alignment was clinically controlled during orthosis fitting to avoid excessive internal or external rotation; however, exact rotational angles were not quantitatively recorded. Retroactive three-dimensional quantification of individual hip joint angles in all anatomical planes was not technically feasible under the clinical bedside setup. However, geometric standardization of the hip position was rigorously maintained via the rigid components of the MGO. All expert adjustments strictly targeted a clinical standard positioning within a therapeutic squatting–spreading target corridor of approximately 100–110° flexion and 40–45° abduction. All orthosis adjustments were performed by experienced clinicians according to established institutional treatment protocols. In cases of unilateral DDH, abduction may be applied selectively to the affected side. Experimental adaptations, such as hemi-Pavlik harness configurations, have also been described in specific clinical contexts [19].

Although the asymmetrical configuration was designed to maximize the range of motion of the unaffected hip, clinical experience suggests reduced parental compliance, primarily due to the visibly asymmetrical posture, which may raise concerns despite its intended therapeutic purpose [10]. Therefore, this study may provide a biomechanical basis for the clinically preferred symmetrical configuration. The observed biomechanical differences should also be interpreted within the broader multifactorial framework of DDH treatment. Clinical treatment success depends not only on orthosis-related force transmission but also on additional determinants such as age at treatment initiation, severity of dysplasia, treatment duration, neuromuscular activity, and parental compliance. Altered axial force transmission within the orthosis may therefore represent only one component influencing therapeutic stabilization and hip maturation. In particular, reduced parental acceptance of asymmetrical orthosis configurations may indirectly affect treatment adherence and overall therapeutic effectiveness.

The following hypotheses were tested:

1. Axial force generation differs between infants with DDH and healthy infants.
2. In infants with unilateral DDH, force generation does not significantly differ between dysplastic and the contralateral healthy hip.
3. In unilateral DDH, axial load does not significantly differ between symmetrical and asymmetrical orthosis configurations.

## 2. Materials and Methods

### 2.1. Study Design and Setting

This study was designed as an exploratory observational cohort study with a comparative approach between healthy newborns and infants with DDH. Participants were recruited consecutively at the Department of Orthopedics, University Medical Center Rostock, between 2013 and 2015.

Ethical approval was obtained from the Ethics Committee of the University Medical Centre Rostock (Approval No. A 48-2008).

### 2.2. Participants

Inclusion criteria comprised newborns aged between 12 h and seven days, with a body weight ranging from 2800 g to 4300 g. The healthy comparison group was randomly selected. The DDH group included infants diagnosed with hip types IIc, D, and IIIa (without limitation of abduction) according to the Graf classification.

For inclusion in the DDH group, the contralateral hip was required to be free of pathological findings. Infants presenting abnormalities in the contralateral hip were excluded from statistical analysis.

Exclusion criteria included skeletal malformations (other than DDH in the affected cohort), neuromuscular disorders, clavicle fractures, brachial plexus palsies, and absence of parental consent. For analyses focusing on unilateral DDH, only infants with confirmed unilateral dysplasia (Graf types IIc, D, or IIIa) and a normal contralateral hip were included.

### 2.3. Experimental Setup and Force Measurement

**Figure 4:**
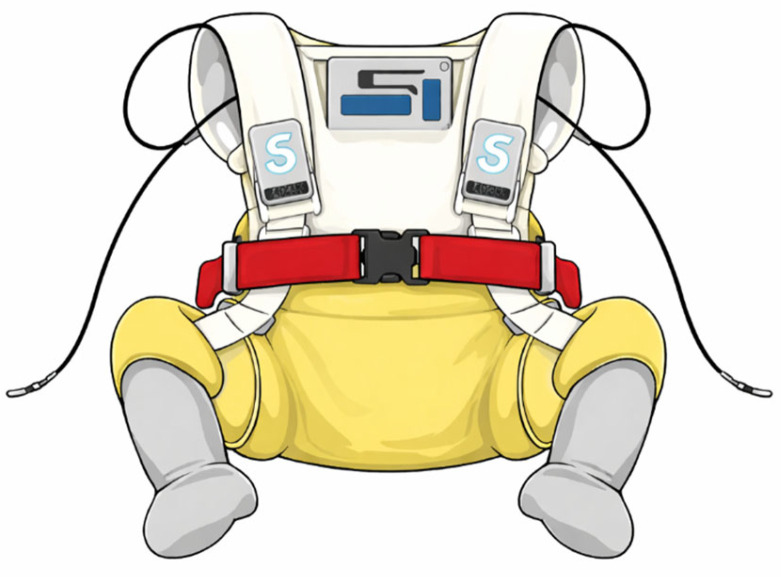
Schematic illustration of sensor integration in the Mittelmeier–Graf hip flexion–abduction orthosis (two sensors in the front, one in the back).

The experimental setup involved integrating three highly sensitive, s-shaped force sensors (Type “KD24s”, ME-MeßSysteme, Hennigsdorf, Germany) into the orthosis’s shoulder straps (Figure 4). These sensors were used without affecting the length of the shoulder straps. Overall, the construction was intended to ensure adequate adjustment even for very small infants. Force transmission was assessed by equipping each strap (front right, front left, and the combined posterior strap) with an individual sensor. All sensors were identical in design and marked clearly. During all measurements, each sensor was placed in its defined location, ensuring the reproducibility of the measurements. All orthosis fittings and sensor placements were performed according to standardized clinical procedures to ensure consistent positioning during measurements. Measurements were conducted under routine clinical conditions with infants positioned in the orthosis in a calm resting state whenever possible. Force sensors were subject to software-based zero-calibration under absolute no-load conditions immediately prior to positioning each neonate within the orthosis. Force data acquisition was performed continuously at a sampling frequency of 50 Hz. To reduce baseline physiological noise and spontaneous minor movement artifacts without loss of macro-biomechanical trends, signal processing involved aggregating the raw data into consecutive 10-s averaging blocks for subsequent statistical analysis. Force values were normalized relative to body weight to improve interindividual comparability. Due to the exploratory pilot character of the study, no advanced filtering algorithms or movement classification procedures were applied beyond temporal averaging into 10-s analysis blocks. The S-shaped sensors are designed for a longitudinal axial tensile and compressive load of up to 100 N. External vertical forces (e.g., from clothing or the examination surface) did not affect the measurements, as confirmed in both the present study and preliminary testing.

### 2.4. Statistical Analysis

Statistical analyses were performed using Microsoft Excel 2017 (Microsoft Corporation, Redmond, WA, USA), IBM SPSS Statistics Version 24 (IBM Corp., Armonk, NY, USA), and R statistical software (R Foundation for Statistical Computing, Vienna, Austria). Data distribution was assessed using the Shapiro–Wilk test. As data in the DDH cohort were not normally distributed, intergroup comparisons were conducted using the Mann–Whitney U test, while paired analyses were performed using the Wilcoxon signed-rank test.

Statistical significance was defined as *p* < 0.05. Given the predefined exploratory comparisons and limited number of statistical tests, no formal correction for multiple testing was applied. Consequently, all *p*-values should be interpreted descriptively and considered hypothesis-generating rather than confirmatory.

No missing data occurred during data collection; all measurements were complete and available for analysis. An a priori power analysis was not performed, as no prior data on axial force transmission in neonatal hip orthoses, particularly distinguishing between healthy infants and those with DDH, were available at the time of study design. Consequently, reliable assumptions regarding effect sizes and variance could not be established.

## 3. Results

### 3.1. Study Population

A total of 36 newborns were enrolled in the study between 2013 and 2015. The participants were divided into two groups:

- Healthy Cohort (*n* = 19): Newborns with sonographically confirmed Type I hips according to Graf. The average age was 2.63 days, and the average weight was 3495 g.
- DDH Cohort (*n* = 17): Newborns diagnosed with unilateral congenital hip dysplasia (Type IIc, D, or IIIa according to Graf). The average age was 2.55 days, and the average weight was 3592 g.

Each measurement period lasted two minutes.

### 3.2. Intergroup Force Comparison (Hypothesis 1)

Table 1 summarizes the descriptive statistics and non-parametric analyses of the cumulative axial forces. The Mann–Whitney U test demonstrated that under the standard symmetrical configuration, the healthy cohort generated significantly higher total axial forces than the DDH cohort (4.02 ± 1.84 N vs. 2.51 ± 1.99 N; *p* = 0.019, r = 0.40). Within the DDH cohort, changing the orthosis from a symmetrical to an asymmetrical configuration resulted in a further descriptive reduction in the mean axial force to 1.83 ± 1.45 N.

**Figure 5:**
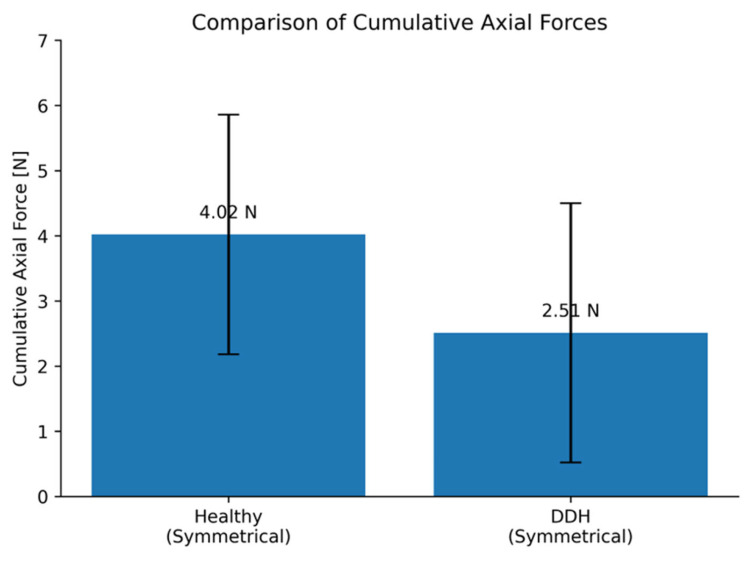
Visual comparison of cumulative axial force generation between healthy infants and infants with DDH under symmetrical orthosis configuration. Bars represent mean total axial force and relative force normalized to body weight.

The detailed descriptive and inferential statistics are presented in Table 1. Healthy infants demonstrated significantly higher cumulative axial forces than infants with DDH under symmetrical orthosis configuration (Figure 5). Within the DDH cohort, asymmetrical orthosis adjustment resulted in descriptively lower mean axial forces, although this difference did not reach statistical significance.

When expressed relative to body weight, axial load in the DDH cohort ranged from 5.20% to 7.13%, whereas the healthy cohort exhibited approximately twofold higher values (11.35% to 11.73%).

### 3.3. Intra-Individual Force Analysis in the DDH Cohort (Hypotheses 2 and 3)

**Figure 6:**
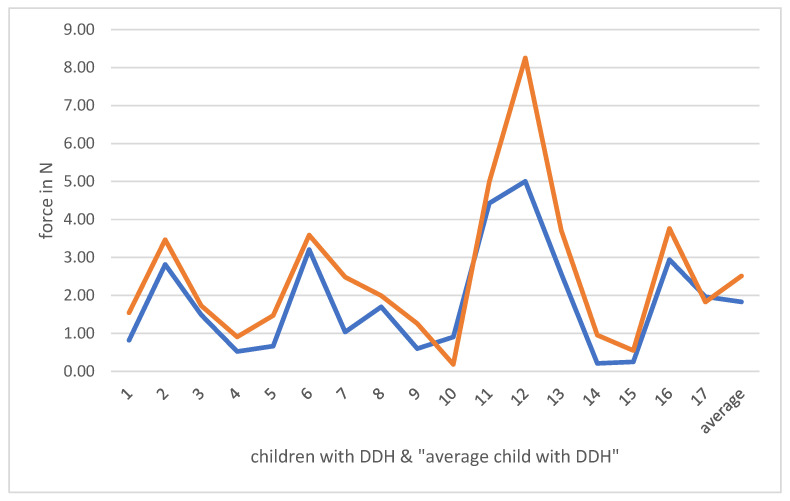
Intra-individual comparison of cumulative axial force transmission in infants with unilateral DDH under symmetrical and asymmetrical orthosis configurations. The blue line depicts the dysplastic hip and the orange line the contralateral healthy hip. The curves represent a single illustrative case and do not correspond to cohort averages.

Within the DDH cohort, axial forces generated by dysplastic hip were compared with those of the contralateral healthy hip (Figure 6). In the symmetrically adjusted orthosis, a non-significant tendency towards lower forces on the dysplastic side was observed (0.82 N vs. 1.05 N; *p* = 0.554). Similarly, in the asymmetrically adjusted configuration, no statistically significant difference in axial load between the treated and untreated side was detected (*p* = 0.356).

Accordingly, neither Hypothesis 2 nor Hypothesis 3 was rejected.

### 3.4. Force Dynamics

**Figure 7:**
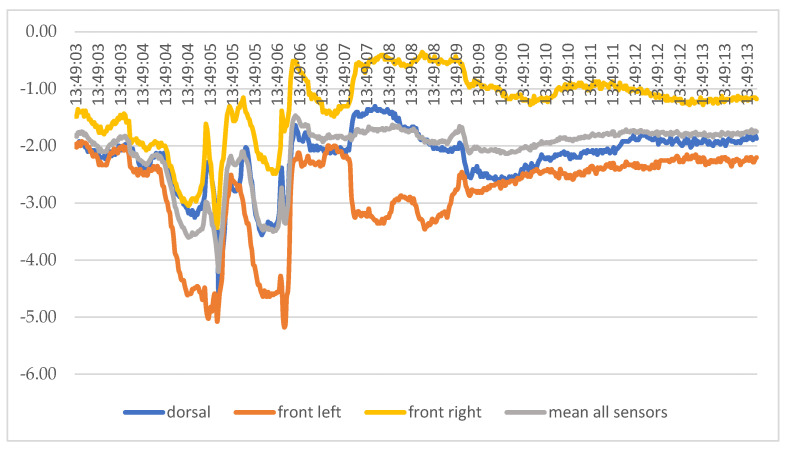
Representative time-dependent force profiles recorded from the three integrated orthosis sensors (front right, front left, dorsal sensor) during spontaneous infant movement. Force fluctuations demonstrate the dynamic character of axial load transmission within the orthotic system. Negative values indicate direction-dependent tensile loading according to sensor orientation.

Force generation was highly dynamic (Figure 7). During quiet periods, forces in the symmetrical setting were low and stable (approx. 1.4–2.5 N). During active phases of kicking, brief force peaks of up to 5.18 N were recorded.

## 4. Discussion

Fetal and neonatal movements generate complex, dynamically varying biomechanical loading conditions that are indispensable for musculoskeletal development and joint maturation [20,21]. Dysplastic hip morphology substantially alters joint biomechanics, including muscle moment arms, joint reaction forces, and load transmission characteristics [22]. Furthermore, infant positioning devices and orthotic systems have been shown to influence muscle activation profiles and spinal loading in early infancy [23]. A recent systematic review on ambulatory bracing confirmed that biomechanical evidence regarding load transmission in orthotic DDH treatment remains limited [24]. Computational biomechanical models have demonstrated that orthotic treatment, such as the Pavlik harness, modifies joint reaction forces and the mechanical environment within the dysplastic hip joint during reduction. However, these models rely primarily on theoretical or simulated load conditions. In contrast, the present study provides experimental in vivo measurements of axial forces generated within a flexion–abduction orthosis, thereby contributing empirical data to the biomechanical understanding of DDH orthotic treatment.

The present findings indicate that infants with DDH exhibited lower axial forces within the orthosis compared to healthy infants. This observation may reflect altered biomechanical or neuromuscular conditions associated with dysplastic hips; however, causal mechanisms cannot be determined from the present exploratory study design. In contrast to computational studies, which primarily rely on simulated loading conditions, the present study provides in vivo experimental data, thereby contributing clinically relevant biomechanical evidence. Although the measured forces do not directly represent intra-articular hip joint reaction forces, they may reflect clinically relevant aspects of orthosis-mediated biomechanical stabilization, neuromuscular tension distribution, and treatment tolerability. However, these results should be interpreted in light of the study’s limitations, including the short measurement duration and relatively small sample size.

In addition, the study aimed to determine whether symmetrical or asymmetrical orthosis adjustment influences axial force transmission. The results suggest that orthosis design may influence axial force transmission within the orthotic system. The findings of the present study should be interpreted in relation to the predefined hypotheses.

**Hypothesis 1.** 
*We hypothesized that axial force generation would not differ between infants with DDH and healthy infants. The results did not support this hypothesis. They demonstrated significantly higher axial forces in healthy infants compared to those with DDH (p ≤ 0.019). This difference may indicate an association between DDH-related biomechanical conditions and altered load generation within the orthotic system. Altered joint stability and muscle mechanics in dysplastic hips may contribute to reduced force transmission.*

**Hypothesis 2.** 
*The second hypothesis assumed no difference in force generation between the dysplastic hip and the contralateral healthy hip in infants with unilateral DDH. This hypothesis was not rejected. No statistically significant intra-individual differences were observed. One possible explanation may be the predominantly symmetrical movement patterns typically observed during early neonatal motor activity.*

**Hypothesis 3.** 
*The third hypothesis postulated that orthosis configuration (symmetrical vs. asymmetrical) would not influence axial load distribution. This hypothesis could not be rejected, as no statistically significant differences were observed between configurations. However, this result should be interpreted with caution. The absence of statistical significance may be influenced by the limited sample size and exploratory study design. Subtle biomechanical differences may not have been detectable under the present conditions. While the asymmetrical setting is biomechanically sound and offers the theoretical benefit of a natural range of motion for the healthy hip, it has proven problematic in clinical practice. The resulting crooked-appearing posture of the infant, though medically sound and comfortable, often leads to considerable parental concern. This has led to compliance issues, with parents attempting to “correct” the posture by misadjusting the brace.*

Consequently, the symmetrically adjusted MGO has become the preferred clinical standard. This choice is further supported by the data: the symmetrical setting provides a superior combination of benefits. It ensures high parental acceptance and compliance while maintaining a biomechanically favorable low-load environment for the infant’s axial skeleton, especially when compared to higher-load designs like the Tübinger splint (mean axial force of 4.09 N in the Superior orthosis vs. 15.1 N in the Tübinger) [14,15]. The main reason for this is the different design in terms of the support function of the legs. This difference stems from their fundamentally distinct approaches to load management. Although both orthoses maintain the desired flexion–abduction positioning, the Mittelmeier–Graf design uses an abdominal belt to absorb axial forces. In contrast, the Tübinger splint design transmits these forces directly to the infant’s shoulders. Treatment duration and clinical outcome in conservative DDH management depend on several factors, including initial severity and patient compliance [14,15]. The observed biomechanical differences should be interpreted within the multifactorial therapeutic context of DDH management, in which treatment success is additionally influenced by dysplasia severity, timing of treatment initiation, treatment duration, muscular adaptation, and parental compliance. Continuous therapy is essential for successful hip maturation and is highly dependent on parental adherence. However, this compliance may be compromised by the asymmetrical configuration of the MGO. Although biomechanically sound, the resulting visibly asymmetrical posture may raise parental concerns, particularly regarding perceived spinal alignment, and thereby reduce acceptance. While both orthosis configurations provide a low axial load environment, this psychological barrier may limit the practical applicability of the asymmetrical setting. The present findings provide clinically relevant insight into orthosis selection: as no biomechanical advantage of asymmetrical adjustment was observed, symmetrical configuration may represent the more practical and clinically applicable treatment approach, particularly in light of improved parental acceptance.

The present findings may have clinical relevance for conservative DDH treatment by improving understanding of biomechanical load transmission within neonatal hip orthoses. More symmetrical and standardized orthosis adjustment may contribute to optimized therapeutic positioning while minimizing unnecessary force transmission. Furthermore, objective biomechanical assessment may support future orthosis optimization, individualized treatment strategies, and improved parental handling and compliance.

### 4.1. Limitations

Several limitations must be acknowledged. First, the sample size is limited, and no a priori power analysis was conducted due to the absence of prior empirical data on axial force transmission in neonatal hip orthoses. In addition, the single-center design may further limit the generalizability of the findings. Consequently, the study should be interpreted as exploratory in nature. The limited cohort size may reduce statistical power and increase the risk of type II error; therefore, the findings should primarily be interpreted as hypothesis-generating rather than confirmatory.

Second, measurements were restricted to short observation periods and therefore represent only a snapshot of loading conditions rather than cumulative exposure during routine orthosis use.

Third, force assessment was performed indirectly via sensors integrated within the orthosis rather than through direct measurement at the level of the hip joint. While this approach ensures methodological consistency and reproducibility, it may not fully capture joint reaction forces. Furthermore, the absence of complementary assessments—such as electromyography or motion analysis—limits insight into the underlying neuromuscular mechanisms.

Potential confounding factors include infant activity level, spontaneous movement patterns, positioning during measurements, behavioral state, and minor differences in orthosis fitting. Although all measurements were performed under standardized clinical conditions with consistent sensor placement and were preferentially restricted to calm resting phases, residual variability related to spontaneous neonatal movement dynamics cannot be fully excluded. A specific limitation of this exploratory study is the absence of synchronized quantitative movement tracking, such as video-based actigraphy, motion capture, or electromyographic assessment, which could have enabled objective covariate analysis of neuromuscular activity and movement intensity. Due to the limited sample size and exploratory pilot character of the study, additional multivariable covariate analyses were considered statistically underpowered and were therefore not performed. Future studies should incorporate synchronized multimodal biomechanical monitoring to better characterize the interaction between movement behavior and orthosis-related force transmission.

Although data acquisition was conducted between 2013 and 2015, the fundamental biomechanical principles governing neonatal movement and load transmission remain unchanged. In the absence of comparable in vivo datasets, the present findings retain their scientific and clinical relevance.

Notwithstanding these limitations, statistically significant intergroup differences were observed, supporting the potential biomechanical relevance of the measured force transmission patterns. Accordingly, the present findings should be interpreted as preliminary biomechanical observations requiring confirmation in larger prospective studies.

### 4.2. Future Directions

Future studies should include prolonged monitoring periods, larger multicenter cohorts, and multimodal biomechanical assessment combining force measurements with electromyography, motion capture, and computational joint-force modeling. Extended recordings under more natural movement conditions may further improve understanding of dynamic load transmission and neuromuscular contributions during conservative DDH treatment.

Orthosis designs may benefit from clearly visible and easy-to-use alignment markers as well as adjustable components that support symmetrical positioning while preserving the therapeutic flexion–abduction configuration required for DDH treatment. Notably, the MGOabduction orthosis already incorporates a traffic-light–style guidance system with red, yellow, and green zones on the abdominal belt. The green zone indicates the recommended therapeutic flexion and abduction positioning and may facilitate standardized orthosis adjustment and parental handling.

The integration of modular and miniaturized sensor systems may enable long-term biomechanical monitoring and provide additional insight into dynamic loading conditions during conservative DDH treatment in everyday clinical settings.

## 5. Conclusions

This exploratory in vivo study indicates that axial load transmission in a hip flexion–abduction orthosis is low and may differ between healthy infants and infants with DDH. Infants with DDH exhibited lower axial forces than healthy newborns, which may reflect differences in biomechanical conditions. As no significant differences were observed between symmetrical and asymmetrical configurations, and considering clinical aspects such as parental compliance, symmetrical adjustment may be preferred in routine practice. Further studies with larger cohorts and extended measurement protocols are required.

## Figures and Tables

**Table 1:** Biomechanical load profiles and non-parametric statistical analyses of cumulative axial forces.

Analysis Level	Orthosis Configuration	Cohort	N	Mean ± SD [N]	Median [N]	95% Confidence Interval [N]	Min–Max [N]	*p*-Value	Effect Size (r)
Intergroup Comparison (Healthy vs. Pathological)	Symmetrical (SS Total H) Symmetrical (SS Total D)	Healthy DDH	19 17	4.02 ± 1.84 2.51 ± 1.99	3.94 1.83	3.13–4.90 1.48–3.53	0.84–7.28 0.18–8.25	0.019	0.40 (Moderate)
Intragroup Comparison (Configuration Effect)	Symmetrical (SS Total D) Asymmetrical (SA Total D)	DDH DDH	17 17	2.51 ± 1.99 1.83 ± 1.45	1.83 1.49	1.48–3.53 1.08–2.58	0.18–8.25 0.21–5.00	0.356 *	—

Note: The paired intra-individual comparison within the DDH cohort was performed using the Wilcoxon signed-rank test. * *p*-value derived from the Wilcoxon signed-rank test.

## Data Availability

The data presented in this study are available on request from the corresponding author. The data are not publicly available due to privacy and ethical restrictions, as they contain potentially identifiable clinical information from pediatric study participants.
